# A pseudo-outbreak of MRSA due to laboratory contamination related to MRSA carriage of a laboratory staff member

**DOI:** 10.1186/s13756-022-01207-7

**Published:** 2023-01-05

**Authors:** Karlijn M. G. Houkes, Joep J. J. M. Stohr, Karin B. Gast, Karen Couderé, Veronica Weterings, Anne Mutsaers - van Oudheusden, Anton G. M. Buiting, Jaco J. Verweij

**Affiliations:** 1 Microvida, Laboratory of Medical Microbiology and Immunology, Elisabeth-TweeSteden Hospital, Tilburg, The Netherlands; 2 Department of Infection Prevention, Amphia Hospital, Breda, The Netherlands; 3 Department of Infection Prevention, Elisabeth-TweeSteden Hospital, Tilburg, the Netherlands; 4 Present Address: Reinier de Graaf Hospital, Delft, The Netherlands

**Keywords:** MRSA, Pseudo-outbreak, Contamination, wgMLST

## Abstract

**Background:**

Methicillin resistant *Staphylococcus aureus* (MRSA) is a major burden for hospitals globally. However, in the Netherlands, the MRSA prevalence is relatively low due to the ‘search and destroy’ policy. Routine multiple-locus variable-number of tandem repeat analysis (MLVA) of MRSA isolates supports outbreak detection. However, whole genome multiple locus sequence typing (wgMLST) is superior to MLVA in identifying (pseudo-)outbreaks with MRSA. The present study describes a pseudo-outbreak of MRSA at the bacteriology laboratory of a large Dutch teaching hospital.

**Methods:**

All staff members of the bacteriology laboratory of the Elisabeth-TweeSteden hospital were screened for MRSA carriage, after a laboratory contamination with MRSA was suspected. Clonal relatedness between the index isolate and the MRSA isolates from laboratory staff members and all previous MRSA isolates from the Elisabeth-TweeSteden hospital with the same MLVA-type as the index case was examined based on wgMLST using whole genome sequencing.

**Results:**

One of the staff members was identified as the probable source of the laboratory contamination, because of carriage of a MRSA possessing the same MLVA-type as the index case. Eleven other isolates with the same molecular characteristics were found in the database, of which seven were retrospectively suspected of contamination. Clonal relatedness was found between ten isolates, including the isolate found in the staff member and the MRSA found in the index patient with a maximum of eleven alleles difference. All isolates were epidemiologically linked through the laboratory staff member, who had worked on all these cultures.

**Conclusions:**

The present study describes a MRSA pseudo-outbreak over a 2.5-year period due to laboratory contamination caused by a MRSA carrying laboratory staff member involving nine patients. In case of unexpected bacteriological findings, the possibility of a laboratory contamination should be considered.

**Supplementary Information:**

The online version contains supplementary material available at 10.1186/s13756-022-01207-7.

## Introduction

*Staphylococcus aureus* is a major human pathogen and an important cause of nosocomial and community-acquired infections [1]. Since the 1960s, methicillin resistant *S. aureus* (MRSA) strains have emerged. These strains harbor a mecA gene making them resistant to almost all β-lactam antibiotics [2, 3]. In the Netherlands, the prevalence of MRSA carriage is low, ranging from 0.03% to 0.17% [4]. Despite this low MRSA prevalence in the Netherlands, nosocomial outbreaks do occur [5]. To detect the source and route of transmission in hospital outbreaks, epidemiological investigation can be combined with molecular typing of the bacterial isolates. Molecular typing of *S. aureus* can be done using Staphylococcal protein A (spa) typing, pulsed-field gel electrophoresis, multiple loci variable number tandem repeat analysis (MLVA), or whole genome multi-locus sequence typing (wgMLST) [6]. The latter has the highest discriminatory power due to the many alleles included in the analysis to identify or dismiss clonal relatedness.

In March 2019, an unexpected MRSA finding in a patient led to the suspicion of a laboratory contamination. This patient had a *S. aureus* infection of a prosthetic joint of the knee. The infection was diagnosed based on methicillin sensitive *S. aureus* (MSSA) in five of eight tissue cultures of the knee. Unexpectedly, MRSA colonies were found in one of the eight cultures. To verify this finding, all original tissue samples were cultured again and swabs originating from the patient’s anterior nares, throat and perineum were cultured to test for MRSA carriage. In none of these cultures, MRSA was found, suggesting that the previously cultured MRSA was a laboratory contamination rather than an actual MRSA infection. For this reason, contact investigation was not performed for the patient’s contacts and infection control measures were lifted. Multiple studies have described laboratory contamination of clinical specimens though various causes [8–10]. The objective of the present study was to determine the source and the extent of this MRSA contamination. Whole genome multiple locus sequence typing (wgMLST) was performed to identify a pseudo-outbreak of MRSA due to laboratory contamination.

## Methods

### Setting and routine microbiology methods regarding MRSA

#### Setting

The Elisabeth-TweeSteden hospital, Tilburg, the Netherlands is a teaching hospital with 796 beds. Around 85 new cases of MRSA carriage or infection are identified each year. Upon hospital admission, all patients are screened for risk factors for MRSA carriage using a questionnaire. Such risk factors are recent hospital admission abroad, professional contact with livestock, intensive contact with a MRSA carrier or a stay in a refugee center in the last two months [11]. In case of a high or intermediate risk, swabs are taken to test for MRSA carriage [11]. This screening is part of the ‘search and destroy’ policy in the Netherlands and is followed by strict isolation and treatment of MRSA carriers [11, 12].

#### Routine microbiology methods regarding MRSA

For MRSA carriage screening swabs of the anterior nares, throat, perineum and, if present, catheters, drains and cutaneous lesions were collected using eSwab medium (Copan, Murrieta, USA) [12]. The swabs were inoculated on a chromogenic MRSA2 Brilliance agar (Oxoid Ltd., Basingstoke, UK), on which MRSA isolates appear as blue colonies after overnight incubation at 35 ± 1 °C, and on a blood agar plate as growth control. The remaining eSwab medium was added to Mueller Hinton Broth (BD Diagnostics, Sparks, USA) supplemented with 6.5% sodium chloride. After overnight incubation at 35 ± 1 °C, the broth was inoculated on a chromogenic MRSA2 Brilliance agar. Species determination of presumptive MRSA colonies was performed by matrix-assisted laser desorption/ionization time-of-flight (MALDI-TOF) mass spectrometry (Bruker Daltonics, Leipzig Germany). Antibiotic susceptibility testing was performed of *S. aureus* isolates using either BD Phoenix 100 system (BD Diagnostics, Sparks, USA) or disc diffusion (BD Diagnostics, Sparks, USA) according to EUCAST [13]. An in-house real-time PCR was performed on isolates with a cefoxitin MIC values > 4 mg/L or cefoxitin (30 μg) disc diffusion diameter < 22 mm to confirm the MRSA identification, detecting the *Sa442* DNA fragment [14], *S. aureus* nuclease (nuc) [15], Panton-Valentine leukocidine (PVL) [16], and methicillin resistance genes MecA and MecC [17, 18]. Additionally, in selected samples (e.g., in case of limited patient isolation capacity) direct molecular screening for MRSA presence can be performed using the Xpert® MRSA NxG detection kit (Cepheid, Sunnyvale, USA). For each patient where MRSA was cultured, the isolate was sent to the National Institute for Public Health and the Environment (RIVM) for further genotyping by MLVA as described by Schouls et al. [19].

### Source and extent of laboratory contamination

#### Source of laboratory contamination

Laboratory staff members were screened for MRSA carriage by sampling of the anterior nares, throat and perineum. These samples were cultured as described above.

#### Extend of laboratory contamination

The laboratory data system was searched for all MRSA isolates cultured in the Elisabeth-TweeSteden hospital from January 2008 until May 2019 with the same MLVA-type as the index MRSA isolate. For each of the detected MRSA isolates with an identical MLVA-type, the likelihood of (laboratory) contamination (likely or unlikely) was determined. Contamination with a MRSA isolate was deemed likely if the MRSA isolate was only cultured once and not in any other sample of the same patient.

### Whole genome sequencing (WGS) and wgMLST

The MRSA index isolate, the MRSA isolates from the laboratory staff members, the MRSA isolates detected in the laboratory data system and the control strain ATCC43300 were selected for WGS. WGS was performed using Nextera XT chemistry on a Miseq sequencer (Illumina, San Diego, CA, USA). After error-correction and de novo genome assembly on CLC genomics workbench 20.0.4 (Qiagen, Germantown, MD, USA), the number of allelic differences between the MRSA isolates was determined using the wgMLST tools of Ridom SeqSphere + version 7.7.5 (Ridom GmbH, Munich, Germany). A total of 2574 alleles were included in the pairwise comparison, in which missing values were ignored. For data visualization, a neighbor-joining tree was created. A maximum allelic difference of 24 alleles was used to identify clusters [20].

## Results

### Source of laboratory contamination

All 23 laboratory staff members working in the bacteriology department were screened for MRSA carriage. Three cultures from two staff members were positive for MRSA. Strain Msta02 was cultured from the perineum of technician 1 and belonged to the MLVA type MT0398-MC0398. Strain Msta03 was cultured from the anterior nares and throat of technician 2 and belonged to MLVA-type MT0489-MC0022, identical to the MLVA-type of the index MRSA isolate Msta01 (Table 1).

**Table 1:** Data of all MRSA isolates found in the Elisabeth-TweeSteden hospital laboratory belonging to MLVA type MT0489-MC0022 from 2008-mid 2019 and the MRSA isolates found by screening the technicians

Isolate ID	Patient/Technician	Sampling date	Material	Clinical MRSA infection	Carriage screening cultures			Direct PCR of sample	Subsequent screening culture	Risk factors for MRSA carriage	Suspect of contamination	MLVA type
					Nose	Throat	Perineum					
Msta01	Patient A (index)	Mar 2019	Synovial fluid / tissue knee	No	–	–	–	-	N/P	None	Yes	MT0489-MC0022
Msta02	Technician 1	May 2019	Perineum	No	–	–	+	N/P	+	None	N/A	MT0489-MC0022
Msta03	Technician 2	May 2019	Nose, throat	No	+	+	–	N/P	+	None	N/A	MT0398-MC0398
Msta04	Patient B	Nov 2016	Nose	No	+	–	–	+	+	Hospital abroad	No	MT0489-MC0022
Msta05	Patient C	Jan 2017	Wound swab	Yes	+	+	+	N/P	+	None	No	MT0489-MC0022
Msta06	Patient D	Apr 2017	Throat	No	–	+	–	–	–	Hospital abroad	Yes	MT0489-MC0022
Msta07	Patient E	May 2017	Skin swab	Yes	+	+	+	N/P	–	Partner of patient C	No	MT0489-MC0022
Msta08	Patient F	Aug 2017	Throat	No	N/P	+	+	N/P	–	Roommate MRSA positive	No	MT0489-MC0022
Msta09	Patient G	Sept 2017	Nose	No	+	–	–	N/P	–	Unknown	Yes	MT0489-MC0022
Msta10	Patient H	Oct 2017	Perineum	No	–	–	+	N/P	–	Contact MRSA positive person	Yes	MT0489-MC0022
Msta11	Patient I	Dec 2017	CAPD dialysate	No	–	–	–	N/P	N/P	None	Yes	MT0489-MC0022
Msta12	Patient J	Mar 2018	Skin swab	No	N/P	N/P	N/P	N/P	N/P	None	ND	MT0489-MC0022
Msta13	Patient K	Apr 2018	Ascites	No	–	–	–	N/P	–	None	Yes	MT0489-MC0022
Msta14	Patient L	Feb 2019	Perineum	No	–	–	+	–	–	None	Yes	MT0489-MC0022

*CAPD* continue ambulante peritoneaal dialyse; *ND* not determined; *N/P* not performed; *N/A* not applicable

### Extent of laboratory contamination

Between January 2008 and May 2019, MLVA-typing was performed on 1037 MRSA isolates. Among those, 12 isolates belonged to the MLVA-type MT0489-MC0022 (including Msta01) and carried a MecA gene. All 12 isolates were found between November 2016 and March 2019 (Table 1) (Fig. 2). Seven of the twelve isolates were suspect for contamination based on the selection criteria (MRSA detected in only 1 sample), namely Msta01 (index patient), Msta06, Msta09, Msta10, Msta11, Msta13, and Msta14 (Table 1). All these isolates were epidemiologically linked through the laboratory staff member Technician 2, who worked on all these cultures. Msta04, Msta05, Msta07, and Msta08 were not suspect for laboratory contamination, since these isolates were found in multiple samples (Table 1). There was no sufficient data to determine the likelihood of contamination of Msta12. There is an epidemiological relationship between the patient C and E since patient E is the partner of patient C. No epidemiological link was detected between any of the other patients.

### Whole genome sequencing and wgMLST

**Fig. 1:**
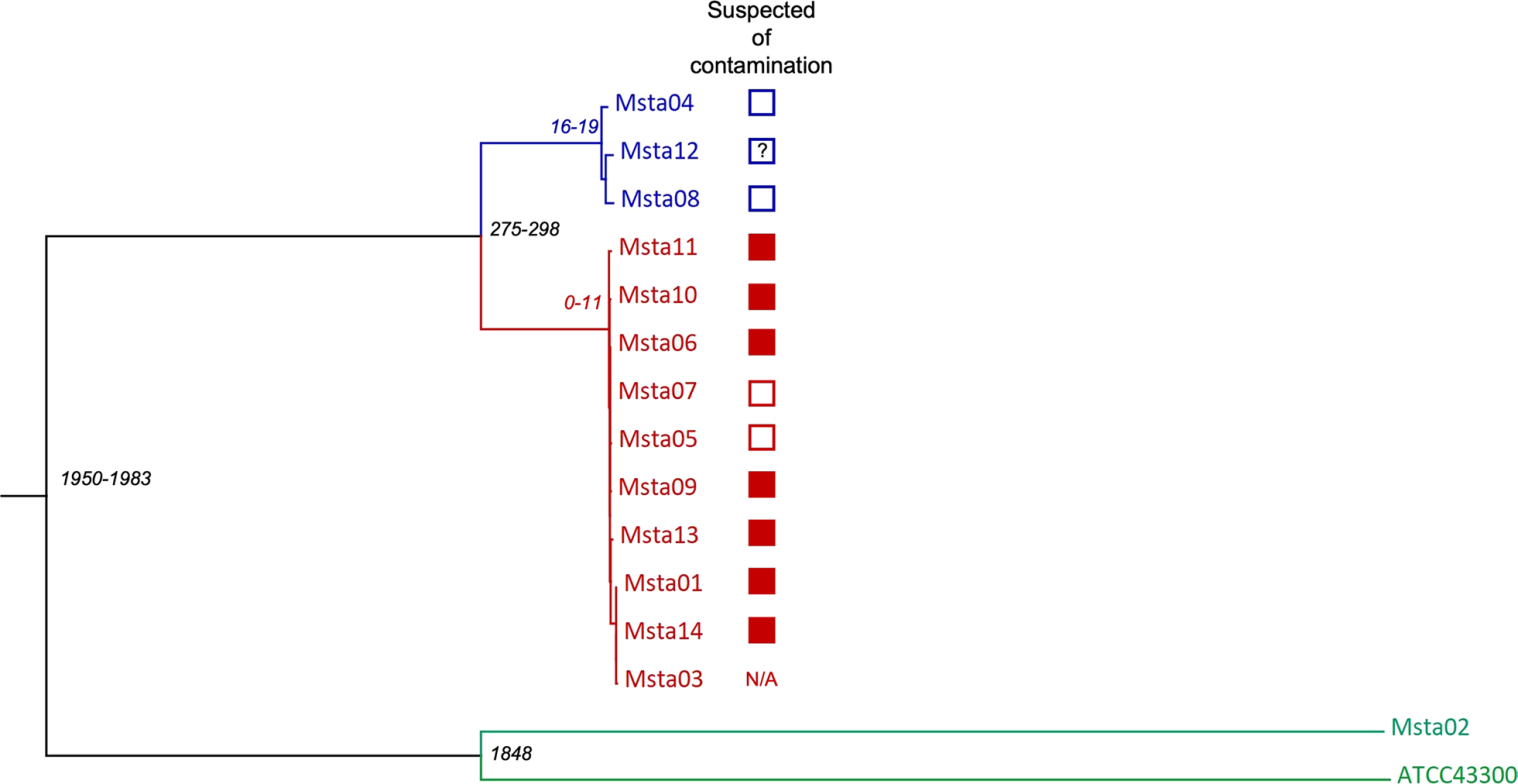
Neighbor-joining tree of MRSA isolates based on wgMLST. The horizontal distance corresponds to the absolute number of allelic differences between isolates. Details of the isolates are depicted in Table 1. The pseudo-outbreak cluster is indicated in red with a maximum allelic difference of 11 alleles. The isolates in blue do have the same MLVA characteristics, but form a separate cluster. Green indicates the MRSA isolates with other MLVA characteristics, including control strain ATCC43300. The number of allelic differences (or range) between clusters are indicated in black and within clusters in red or blue corresponding to the cluster color

**Fig. 2:**
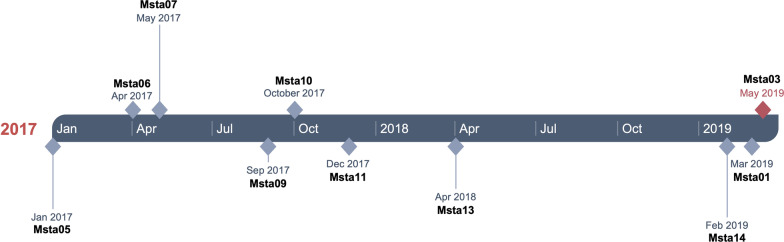
Timeline of the pseudo-outbreak cluster due to laboratory contamination. The MRSA isolates belonging to the cluster are indicated with a diamond shape on the point in time when the isolate was first cultured. The MRSA isolate found in the staff member with the same MLVA type is indicated in red

Whole genome sequence data was generated for all isolates described in Table 1 and the ATCC43300 reference strain. All assembled genomes met the quality criteria (Additional file 1: Table S1). WgMLST revealed two clusters (Fig. 1). The cluster indicated in red in Fig. 1 consists of Msta03, detected in technician 2, Msta01, Msta05, Msta06, Msta07, Msta09, Msta10, Msta11, Msta13 and Msta14. The number of alleles difference between these 10 isolates ranged from 0 to 11 alleles, indicating that they belong to the same genetic cluster (Additional file 2: Table S2). Within this cluster, 8 isolates (Msta01, Msta03, Msta06, Msta09, Msta10, Msta11, Msta13 and Msta14) were suspected of laboratory contamination. However, Msta05 and Msta07 were not suspected of laboratory contamination. The timeline of the identified pseudo-outbreak cluster revealed that, chronologically, the outbreak starts with Msta05 (Fig. 2). The second cluster is indicated in blue in Fig. 1 and consists of Msta04, Msta08 and Msta12 with a difference ranging from 16 to 19 alleles (Additional file 2: Table S2). None of these isolates was suspected of laboratory contamination and epidemiological links were absent in this cluster. The isolates in the second cluster differed at least 275 alleles from the first identified cluster containing both Msta01 (index isolate) and Msta03 found in technician 2.

## Discussion

This report describes a MRSA pseudo-outbreak due to a laboratory contamination by a MRSA carrying laboratory staff member involving nine patients over a period of 2.5 years. The pseudo-outbreak cluster was identified by wgMLST and had a maximum allelic difference of 11 alleles. A previous wgMLST cluster analysis study found a relatedness threshold of < 24 alleles for *S. aureus* [20]. However, all difference > 5 alleles should be interpreted with caution and in relation to the presence or absence of an epidemiological link [21]. Moreover, the determination of the relatedness threshold of wgMLST is complicated by the evolution rate of active growing of isolates, which is 1 mutation per 6 weeks in the case of MRSA [22]. In the present study, two clusters were identified. One is the pseudo-outbreak cluster in which the most divergent samples within the cluster were isolated 17 months apart, which could explain the increased number of allelic differences. The two isolates isolated in the last three months of the pseudo-outbreak differ only 1 allele from the isolate found in the staff member. Furthermore, all isolates in this cluster had an epidemiological link through the MRSA carrying staff member. Based on wgMLST and their epidemiological link, it is likely that all nine MRSA isolates do belong to the pseudo-outbreak. The other cluster consisting of three MRSA isolates without an epidemiological link, are not part of an (pseudo-)outbreak based on this analysis. Although these isolates had the same MLVA-typing, based on wgMLST these three isolates were clearly distinct from the isolates belonging to the pseudo-outbreak cluster. This illustrates the added value of wgMLST compared to MLVA-typing.

Chronologically, the first isolate of the pseudo-outbreak cluster was not suspected of contamination, since the MRSA carriage in this patient was confirmed by multiple cultures making laboratory contamination highly unlikely. The laboratory staff member may have been infected with Msta03 during culturing of Msta05 in January 2017. It is only after January 2017 that we observed an increase of MRSA isolates in the Elisabeth-TweeSteden hospital with MLVA MT0489-MC0022. The only MRSA isolate with the same MLVA-type isolated before 2017 (Msta04) did not belong to the same cluster according to the WgMLST analysis. The MRSA carrying staff member had no risk factors for MRSA carriage. Although we have no definite proof, it seems most likely that the laboratory staff member was infected during laboratory activities. Infections acquired during laboratory work with various other bacteria have been described, but MRSA is not recognized as a pathogen that presents a risk of laboratory infection [23]. An increased incidence for *Staphylococcus aureus* carriage was found in a Dutch cross-sectional study among laboratory staff members, but observed a MRSA prevalence comparable to that of the general population [24]. In the present study, two of the 23 laboratory staff members working at the bacteriology department were MRSA carriers (8.9%) (unrelated strains). This is more than could be expected based on the general Dutch population where the MRSA prevalence is < 1% [4, 12]. However, more research is needed to determine whether there is an increased risk for MRSA carriage among laboratory staff members.

Although pseudo-outbreaks due to laboratory contamination of clinical specimens have been reported [7–10], to the best of our knowledge, no pseudo-outbreak due to MRSA carriage of a laboratory staff member has been described before. This may be due to reporting bias, but also due to a lack of awareness recognizing such pseudo-outbreaks. At the time the current pseudo-outbreak due to laboratory contamination was detected, clinical specimens were inoculated manually. It is likely that contamination occurred during inoculation or handling the culture plate after initial incubation. Automated specimen processing could minimize the risk of contamination. To enable early detection of pseudo-outbreaks, whole genome sequencing of newly identified MRSA isolates could be performed routinely in search for clusters within the laboratory specific database. We recommend to further investigate clusters without an epidemiological link and to consider screening laboratory employees when laboratory contamination is suspected. Further investigation into MSSA and MRSA carrying laboratory staff members using wgMLST could provide more evidence on the possible relationship between MSSA and MRSA carriage and microbiological laboratory work.

## Conclusion

A pseudo-outbreak of MRSA was identified involving nine patients caused by MRSA carriage of a laboratory staff member who contaminated clinical specimens. Clonal relatedness between the samples suspected of contamination could be confirmed by wgMLST, showing the added value over MLVA-typing. This pseudo-outbreak emphasizes the importance of critical and continuous evaluation of microbiology laboratory procedures to minimize the possibility of laboratory contamination and to maximize early detection of false-positive culture results.

## Supplementary Information

**Additional file 1. Supplementary table 1.** Quality control values of whole genome sequencing.

**Additional file 2. Supplementary table 2.** Distance matrix of the wgMLST analysis of the MRSA strains from the laboratory database with MLVA type complex MC0022, MLVA type MT0489 and MLVA profile 18-05-03-01-01-13-01-05 and the two medical microbiology technicians tested positive for MRSA in the pseudo-outbreak investigation. The colors of the isolate IDs and the colored absolute number of allelic differences correspond to the cluster they belong to as depicted in Figure 1.

## Data Availability

Genomic sequences are available under the NCBI BioProject accession number PRJEB58118.
