# Supplementary figures and images for: A pseudo-outbreak of MRSA due to laboratory contamination related to MRSA carriage of a laboratory staff member
Source: Antimicrob Resist Infect Control. 2023 Jan 5;12:1. doi: 10.1186/s13756-022-01207-7 (PMC9814305; doi:10.1186/s13756-022-01207-7)

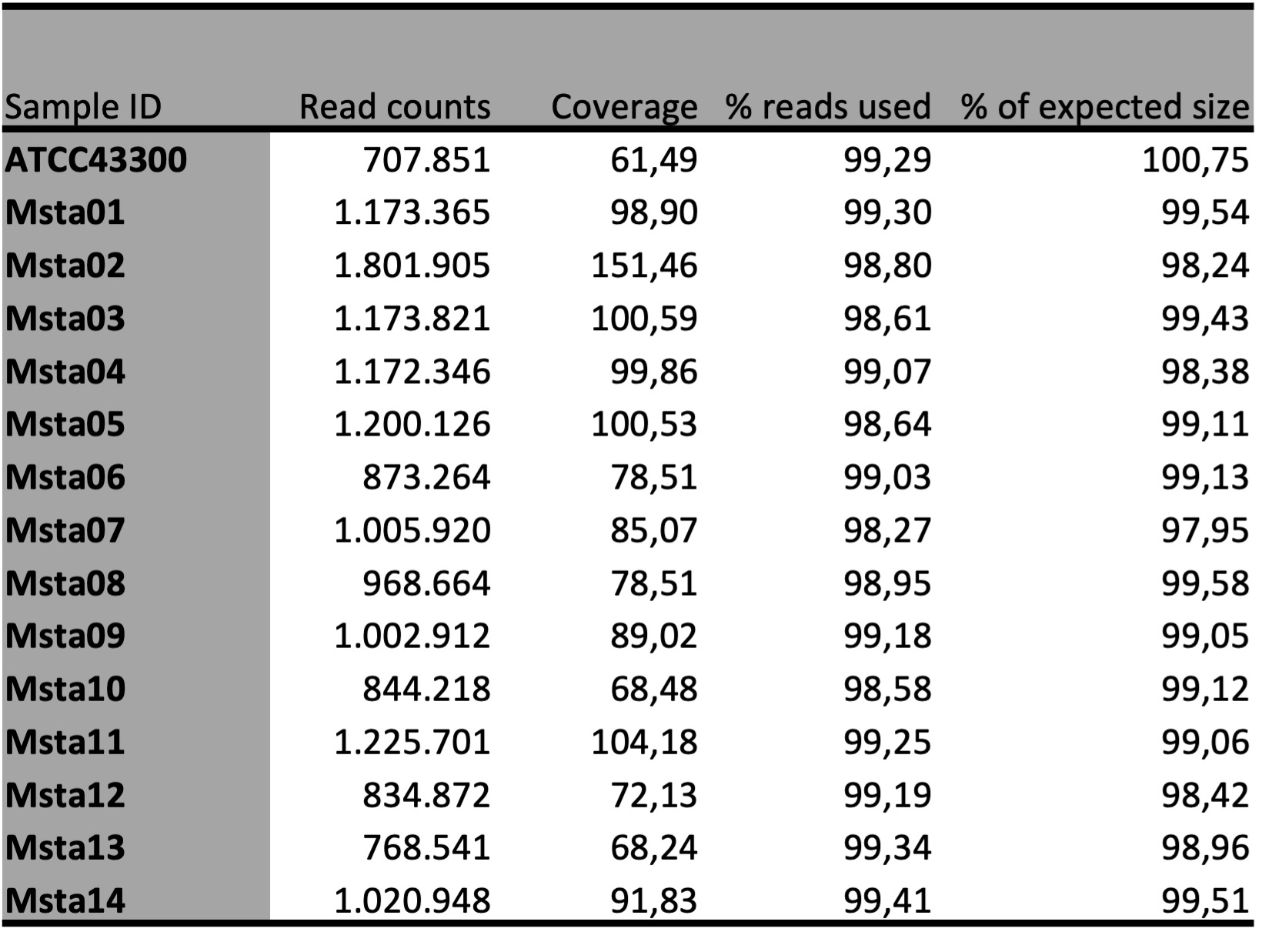

Supplement: Supplementary file 1 — Additional file 1. Supplementary table 1. Quality control values of whole genome sequencing. [file 13756_2022_1207_MOESM1_ESM.jpg]

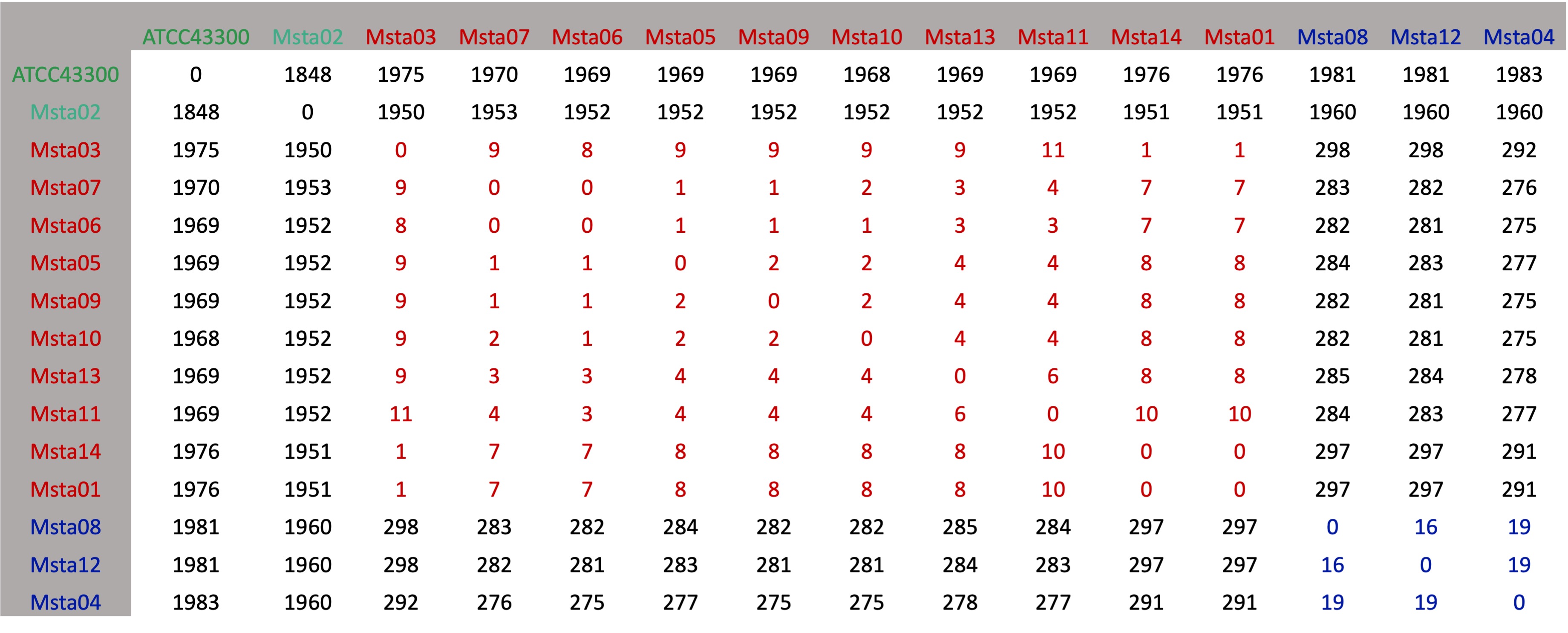

Supplement: Supplementary file 2 — Additional file 2. Supplementary table 2. Distance matrix of the wgMLST analysis of the MRSA strains from the laboratory database with MLVA type complex MC0022, MLVA type MT0489 and MLVA profile 18-05-03-01-01-13-01-05 and the two medical microbiology technicians tested positive for MRSA in the pseudo-outbreak investigation. The colors of the isolate IDs and the colored absolute number of allelic differences correspond to the cluster they belong to as depicted in Figure 1. [file 13756_2022_1207_MOESM2_ESM.jpg]
